# A biophysical mechanism for preferred direction enhancement in fly motion vision

**DOI:** 10.1371/journal.pcbi.1006240

**Published:** 2018-06-13

**Authors:** Alexander Borst

**Affiliations:** Max-Planck-Institute of Neurobiology, Martinsried, Germany; École Normale Supérieure, College de France, CNRS, FRANCE

## Abstract

Seeing the direction of motion is essential for survival of all sighted animals. Consequently, nerve cells that respond to visual stimuli moving in one but not in the opposite direction, so-called ‘direction-selective’ neurons, are found abundantly. In general, direction selectivity can arise by either signal amplification for stimuli moving in the cell’s preferred direction (‘preferred direction enhancement’), signal suppression for stimuli moving along the opposite direction (‘null direction suppression’), or a combination of both. While signal suppression can be readily implemented in biophysical terms by a hyperpolarization followed by a rectification corresponding to the nonlinear voltage-dependence of the Calcium channel, the biophysical mechanism for signal amplification has remained unclear so far. Taking inspiration from the fly, I analyze a neural circuit where a direction-selective ON-cell receives inhibitory input from an OFF cell on the preferred side of the dendrite, while excitatory ON-cells contact the dendrite centrally. This way, an ON edge moving along the cell’s preferred direction suppresses the inhibitory input, leading to a release from inhibition in the postsynaptic cell. The benefit of such a two-fold signal inversion lies in the resulting increase of the postsynaptic cell’s input resistance, amplifying its response to a subsequent excitatory input signal even with a passive dendrite, i.e. without voltage-gated ion channels. A motion detector implementing this mechanism together with null direction suppression shows a high degree of direction selectivity over a large range of temporal frequency, narrow directional tuning, and a large signal-to-noise ratio.

## Author summary

Seeing the direction of motion is essential for survival of all sighted animals. Consequently, nerve cells that respond preferentially to visual stimuli moving in a certain direction are found abundantly. However, directional information is not represented at the level of single photoreceptors but rather has to be computed by subsequent neural circuits. Algorithmic models have been proposed in the past that calculate the direction of motion by multiplying and/or dividing the input signals from neighboring photoreceptors after asymmetric temporal filtering. But how can neurons multiply or divide? Inspired by recent data from fly motion-sensitive neurons, I present a biophysical model of a nerve cell that is based on purely passive conductance changes. I show that such a model can reveal a high degree of direction selectivity over a large range of temporal frequency, narrow directional tuning, and a large signal-to-noise ratio.

## Introduction

Motion represents an essential visual cue, used for predator avoidance, prey capture and visual navigation throughout the animal kingdom. Accordingly, motion-sensitive neurons are found in various brain areas of vertebrates and invertebrates alike. Prominent and well-studied examples are retinal ganglion cells of the rabbit [1,2], retinal starburst cells [3] and ganglion cells of the mouse [4,5], cortical neurons of the mouse [6], cat [7], ferret [8] and monkey [9,10], neurons of the accessory optic system of birds [11] and the lobula plate tangential cells of flies [12–15] (for review, see [16]). To describe the response properties of these neurons, a number of partially equivalent models [17] have been developed, e.g. the Hassenstein-Reichardt detector [18], the Barlow-Levick detector [2], the F-model [19], the elaborated Reichardt model [20] and the energy model [21]. As a common feature, these models compute the local direction of motion by correlating the luminance values of adjacent image pixels after asymmetric temporal filtering. With one exception [22], however, the biophysical implementation of such a correlative, multiplicative-like interaction has so far not been elucidated.

In the fruit fly *Drosophila*, visual signals are processed in the optic lobe, a brain area comprised of the lamina, medulla, lobula, and lobula plate, each arranged in a columnar, retinotopic fashion [23–25] (for review, see [26,27]). In striking parallel to the vertebrate retina [28], the direction of visual motion is computed within the optic lobe separately in parallel ON and OFF motion pathways [29–33]. Within each column, four T4 and four T5 cells represent the local output signals of the ON (T4) and the OFF (T5) channel, each one of them tuned to one of the four cardinal directions projecting accordingly to one of the four layers of the lobula plate [34]. There, T4 and T5 cells provide direct excitatory cholinergic input onto the dendrites of wide-field, motion-sensitive tangential cells [35,36] as well as onto glutamatergic lobula plate interneurons that inhibit wide-field tangential cells in the adjacent layer [37,38]. Electrophysiological [32], optical voltage [39] and Calcium recordings [33,40–43] from presynaptic medulla neurons revealed that none of them is directionally selective. Therefore, T4 and T5 cells are the first neurons in the visual processing chain that respond to visual motion in a direction selective manner [34,44].

**Fig 1:**
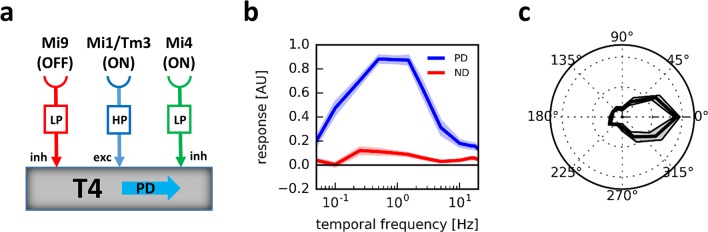
Main input elements and response properties of the direction-selective T4 neuron of the fly. **a** Elementary motion detecting T4 neurons receive input from at least four types of non-directional interneurons, called Mi9 on the preferred side of its dendrite, Mi1 and Tm3 in the center and from Mi4 on the null side of its dendrite (data from [50]). The arrangement shown accounts for a T4 cell with rightward as its preferred direction. For a T4 cell tuned to leftward motion, Mi9 and Mi4 would switch places. **b** Temporal frequency tuning of T4 cells to drifting gratings. Note the high direction selectivity of T4 cells exhibiting almost no response to gratings drifting along the null direction of the cell (Figure reused from [47] under their CC-BY license). **c** Directional tuning of T4 cells. Already for gratings moving at +- 45 degree away from the preferred direction, the response falls off to 50% of maximum response (data from [47]).

Different studies provided evidence that T4 and T5 cells become selective for the direction of motion mainly by preferred direction enhancement [44,45], by null direction suppression only [46], and by a combination of both mechanisms [47–49]. As shown by apparent motion stimuli placed precisely on the hexagonal lattice of the columns via a telescope, individual stimuli interact in a supralinear way on the preferred side of the dendrite, while they suppress each other when delivered on the null side of the dendrite [47,48]. An electron microscopy study revealed that T4 cells receive input on their dendrites from columnar neurons in a topographic order that follows their directional preference [50]: Mi9 cells contact T4 cells’ dendrites on their preferred side, Mi1 and Tm3 cells provide input in the central part, while Mi4, C3 and TmY15 are presynaptic on the null side of the dendrite. Intriguingly, 2-Photon Calcium imaging showed an OFF center receptive field for Mi9, while Mi1, Tm3 and Mi4 all exhibit an ON center [42]. With respect to their temporal filter properties, the same study found Mi9 and Mi4 to be slow and sustained, well described by a temporal low-pass filter. In agreement with previous electrophysiological studies [32], Mi1 and Tm3 turned out to be fast and transient, with temporal band-pass properties [42]. Taken together, the above results suggest that a preferred direction enhancement is realized by a supralinear interaction between Mi9 and the central inputs Mi1 and Tm3 on the preferred side and a null direction suppression by Mi4 on the null side of the T4 cells’ dendrite (Fig 1A). Indeed, multiplying the positive, i.e. sign-inverted Mi9 signal with the one from Mi1 and dividing the result by the one from Mi4 results in a tuning characteristic of the postsynaptic T4 cell that matches the experimental data in detail [42]. In response to gratings drifting along the preferred and null direction at temporal frequencies over two orders of magnitude, the preferred direction response of T4 cells peaks at around 1 Hz while the null direction response is close to zero over the whole range (Fig 1B, [34,47]). In response to gratings drifting along various directions, T4 cells exhibit a rather narrow directional tuning (Fig 1C, [34,47]). A similar model has been proposed in [43]. However, in contrast to the one explained above, this one derives its direction selectivity mainly from a multiplicative interaction between Mi1 and Tm3 impinging on different parts of the dendrite with the result being attenuated by flanking inputs from Mi9 and Mi4 [43].

How should such operations be realized in terms of biophysics of nerve cells? How can neurons multiply or divide, and what is the role of the OFF center receptive field of the enhancing neuron? Here, the following evidence exists with respect to the transmitter phenotype of the various input neurons: (1) Mi9 is immuno-positive for the vesicular glutamate transporter VGluT [50]. Interestingly, in insects, glutamate can also exert hyperpolarizing, inhibitory action on the postsynaptic neuron via the glutamate-gated chloride channel GluClα [51,37,38]. Indeed, the transcript for GluClα has been found in mRNA pooled from T4 and T5 cells [52]. (2) Mi1 and Tm3 are immuno-positive for the acetylcholine-synthesizing enzyme choline-acetyl-transferase (ChAT, [50]) and also express the vesicular acetylcholine-transporter (VAChT, [53]). (3) Mi4 are immuno-positive for the GABA-synthesizing enzyme glutamic-acid-decarboxylase GAD1 [50]. These observations suggest that the two flanking neurons, Mi9 and Mi4, are inhibitory, while the central inputs are excitatory on the postsynaptic T4 cell, and will provide the substrate of a biophysical implementation of the fly motion detector in the ON pathway proposed in the following.

## Results

**Fig 2:**
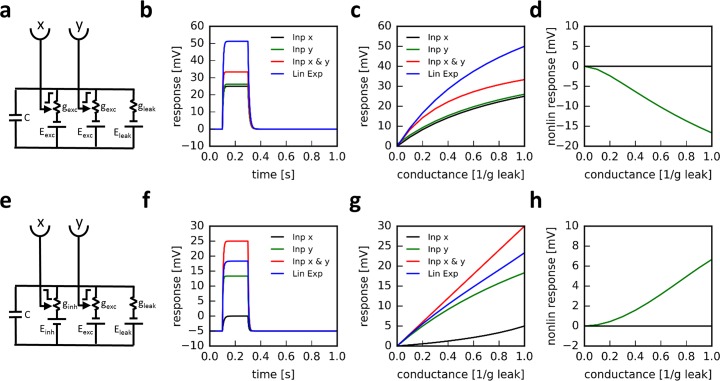
Response properties of passive electrical equivalent circuits. **a** Circuit with two excitatory conductances (*E*_*exc*_ = +50 *mV*). **b** Time course of voltage responses to each input alone, to both inputs simultaneously and the sum of the individual responses (‘linear expectation’). **c** Voltage responses as a function of the input conductance, again for each of the inputs alone, to both inputs simultaneously and the sum of the individual responses. **d** Nonlinear response, defined as the difference between the linear expectation and the responses to the combined inputs as a function of the input conductance. The circuit shows sublinear behavior, i.e. the nonlinear response is always negative. **e-h** Same as above, but for a circuit with one inhibitory and one excitatory conductances (*E*_*exc*_ = +50 *mV*, *E*_*inh*_ = −10 *mV*). Importantly, the left input exerts a negative control on the inhibitory conductance: the larger its input, the smaller the conductance. This results in a supralinear behavior of the membrane voltage, i.e. the nonlinear response is always positive.

Following a previous suggestion [54], I will first describe how even a passive membrane model can reveal positive, multiplicative-like signal amplification. In order to extract the nonlinearity from the circuit, the responses to two inputs delivered simultaneously are compared with the sum of the responses to each individual stimulus presentation (‘linear expectation’). Let us first consider a simple electrical equivalent circuit of a passive isopotential neuron that receives two excitatory input signals x and y acting on the excitatory conductances g_exc1_ and g_exc2_ (Fig 2A).

The steady-state postsynaptic membrane potential V_m_ is given by:

$$V_{m}=\frac{E_{exc}(g_{exc1}+g_{exc2})+E_{leak}g_{leak}}{g_{exc1}+g_{exc2}+g_{leak}}$$ (1)

When we express V_m_ as the difference between V_m_ and E_leak_ and all conductances relative to g_leak_, this becomes:

$$V_{m}=E_{exc}\frac{g_{exc1}+g_{exc2}}{g_{exc1}+g_{exc2}+1}$$ (2)

For *x* = *g*_*exc*1_ and *y* = *g*_*exc*2_, the responses to each individual input are (Fig 2B and 2C):

$${R_{1}=E}_{exc}\frac{x}{x+1};\;{R_{2}=E}_{exc}\frac{y}{y+1}$$ (3)

For x = y, the linear expectation becomes (Fig 2B and 2C)

$${R_{1}+R_{2}=E}_{exc}\left(\frac{2x}{x+1}\right)$$ (4)

The membrane response to both inputs given simultaneously equals (Fig 2B and 2C):

$${R_{1,2}=E}_{exc}\left(\frac{2x}{2x+1}\right)$$ (5)

From this, we calculate the nonlinear response component as the difference between the response to both inputs and the linear expectation (Fig 2D):

$$R_{Nonlin}=E_{exc}\frac{{-2x}^{2}}{2x^{2}+3x+1}$$ (6)

We see that the response of an electrically passive neuron is always sublinear, i.e. the response to the simultaneous activation of two excitatory inputs is smaller than the sum of the responses to each excitatory input individually. Hence, the nonlinear response component is negative and decreases with increasing input signals (Fig 2D).

We next investigate the situation for a combination of an excitatory and an inhibitory input. The membrane potential becomes:

$$V_{m}=\frac{E_{exc}g_{exc}+E_{inh}g_{inh}}{g_{exc}+g_{inh}+1}$$ (7)

Let us now assume that the excitatory conductance follows the input signal x, while the inhibitory conductance follows 1-y (with 0 ≤ *y* ≤ 1), i.e. the inhibitory conductance becomes the smaller the larger the input signal y (Fig 2E).

$$V_{m}=\frac{E_{exc}x+E_{inh}(1-y)}{x+(1-y)+1}$$ (8)

As we will see, this has interesting consequences for the nonlinearity of the postsynaptic membrane voltage. The resting membrane potential, i.e. when x = y = 0, now is *V*_*rest*_ = *E*_*inh*_/2, and, as before, all membrane responses will be expressed relative to this resting potential.

The individual responses become (Fig 2F and 2G)

$$R_{1}=({2E}_{exc}-E_{inh})\frac{x}{2(2+x)};\;R_{2}=(-E_{inh})\frac{y}{2(2-y)}$$ (9)

For x = y, the linear expectation becomes (Fig 2F and 2G)

$$R_{1}+R_{2}=(E_{exc}(2-x)-2E_{inh})\frac{x}{4-x^{2}}$$ (10)

The membrane response to both inputs given simultaneously equals (Fig 2F and 2G)

$$R_{1,2}=(E_{exc}-E_{inh})\frac{x}{2}$$ (11)

From this, the nonlinear response component is calculated as (Fig 2H)

$$R_{Nonlin}=(E_{exc}(2-x)+E_{inh}x)\frac{x^{2}}{2(4-x^{2})}$$ (12)

For 0 ≤ *x* ≤ 1, i.e. positive conductances smaller or equal to the leak conductance, and abs(E_exc_) > abs(E_inh_), this expression is always positive. Therefore, a passive membrane reveals a signal amplification if one of the two inputs decreases an inhibitory input conductance. Intuitively, this is because the input reduces the input resistance of the postsynaptic neuron and therefore leads to an increased response to an excitatory input as compared to when the latter is given in isolation (Fig 2H).

**Fig 3:**
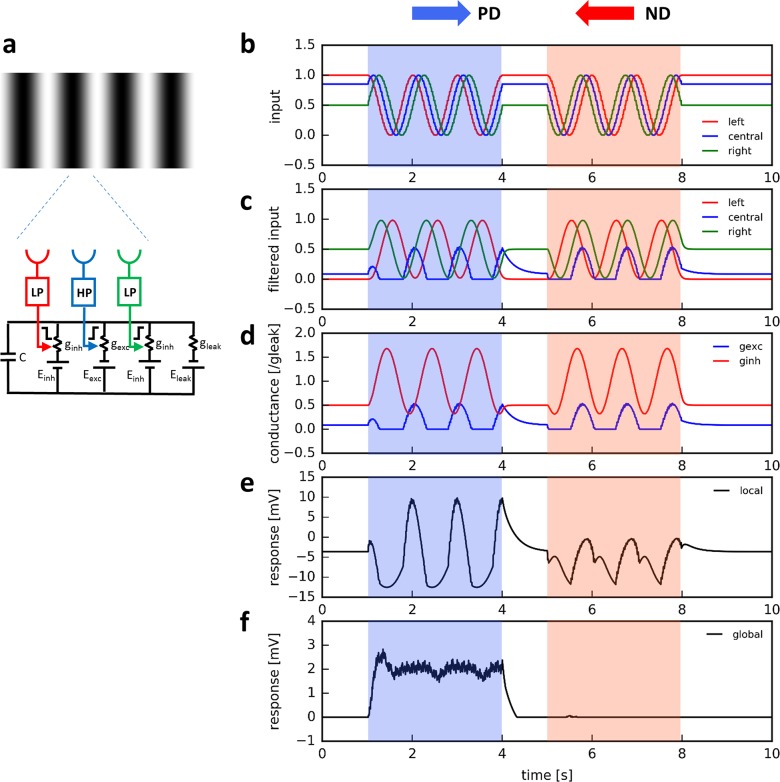
Motion detector together with its internal and output signals in response to a moving sine-grating. **a** The detector builds on a passive electrical equivalent circuit. It receives input from three neighboring locations: the left and the right input become low-pass filtered, the central input becomes high-pass filtered. Importantly, the left input is represented by an OFF cell controlling an inhibitory conductance. Its interaction with the central input implements preferred direction enhancement. The central and the right input are ON units controlling an excitatory and an inhibitory conductance, respectively. This interaction implements a null direction inhibition. **b** Time course of input signals (left, central and right) in response to the drifting sine-grating (1Hz, 100% contrast). **c** Filtered output signals of the left, central and right unit. Note that the left input is inverted, because its properties of an OFF unit. **d** Time course of the excitatory and inhibitory conductance. Note that, during preferred direction motion, the excitatory conductance peaks at the trough of the inhibition. During null direction motion, both conductances peak at around the same time. **e** Resulting membrane potential of the motion detector. **f** Average membrane potential across the population of individual motion detectors. Before averaging, the output signal of each individual detector is rectified at a membrane potential of 0 mV.

In order to explore whether such a biophysical mechanism is indeed useful to extract the direction of motion, I simulated a two-dimensional array of 40 x 40 motion detectors covering a visual space of 180 by 180 deg at a temporal resolution of 10 msec. Each detector received input from 3 neighboring locations in visual space (Fig 3A). Input from the left and right location became processed by a 1^st^ order low-pass filter with a time-constant of 50 msec, while the central input was 1^st^ order high-pass filtered with a 250 msec time-constant, plus a DC component of 10% [30]. These signals acted on an electrical equivalent circuit of a passive piece of membrane. Since the dynamics of the input signals were assumed to be large against the membrane time-constant, the capacitive current could be neglected. Importantly, the left input was simulated as an OFF channel controlling an inhibitory conductance, i.e. this signal became the smaller the larger the local luminance. The central input was treated as an ON channel controlling an excitatory conductance and the right input as an ON channel controlling an inhibitory conductance. This way, preferred direction enhancement, as proposed in the Hassenstein-Reichardt detector [18], was implemented by the interaction between the left and the central input, and null direction suppression, as proposed in the Barlow-Levick detector [2], by the action of the inhibitory right input. Fig 3B–3D show the input signals (Fig 3B), their filtered versions (Fig 3C) as well as the resulting conductances (Fig 3D) in response to a sine-grating moving at 1 Hz along the preferred and the null direction of the motion detector. The peak of the excitatory conductance coincides with the trough of the inhibitory conductance during preferred direction motion and with the peak during null direction motion (Fig 3D). Therefore, the resulting membrane voltage of a single motion detector depolarizes periodically up to 10 mV during preferred direction motion, while it remains hyperpolarized during null direction motion (Fig 3E). In analogy to a lobula plate tangential cell receiving excitatory input from T4 cells [55,37,38], the output voltages of all local motion detectors were rectified at a membrane potential of 0 mV and averaged across the population. This signal reveals maximum direction selectivity with sustained depolarization during preferred direction and zero response to null direction motion (Fig 3F).

**Fig 4:**
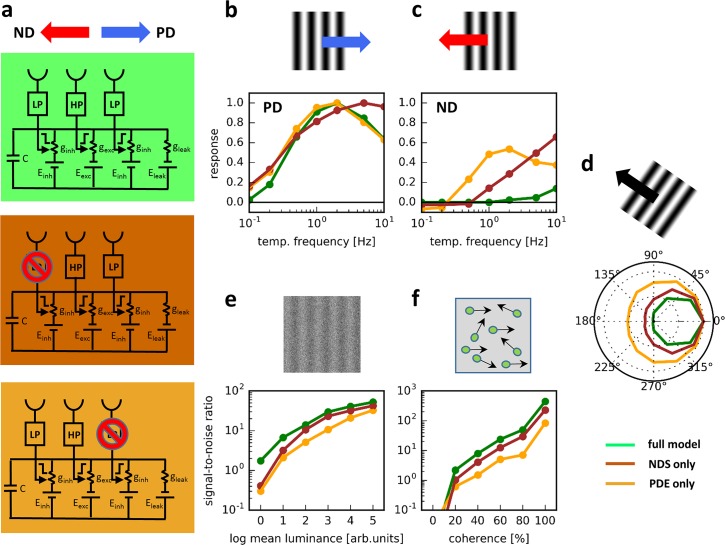
General response properties of the motion detector. **a** The full model (top), together with two partial models where either the left (Null direction suppression = NDS only, middle) or the right input is blocked (Preferred direction enhancement = PDE only, bottom). **b,c** Temporal frequency tuning of all three motion detectors for sine-gratings moving along the preferred (b) and null (c) direction. The responses were normalized to the peak response during preferred direction. **d** Directional tuning of all three motion detectors for sine-gratings moving at 1 Hz. Again, responses were normalized to the peak response. **e** Sensitivity to photon noise of the motion detector response to sine-gratings moving at 1 Hz along the preferred direction. As the mean luminance increases, the relative contribution of photon noise decreases. **f** Signal-to-noise ratio of the motion detector responses to moving dots as a function of the percentage of dots which move along the preferred direction (‘% coherence’).

This motion detector (Fig 4A, top) was tested under various conditions. In a first series of simulations, a sine-grating was used drifting from 0.1 Hz up to 10 Hz along either the preferred (Fig 4B) or the null direction (Fig 4C) of the detector. To assess the directional tuning of the motion detector, the models were next stimulated by a sine-grating, again with 100% contrast, drifting at 1 Hz in various directions in steps of 30 deg (Fig 4D). Finally, I tested the noise susceptibility of the motion detector in two different situations. In one case, photon noise was simulated by presenting a sine-grating first drifting along the preferred and then along the null direction of the motion detector. At each time point, the actual pixel value was drawn from a Poisson distribution with a mean value λ proportional to the value of each image pixel independently (Fig 4E; for details, see Methods). Different noise levels were achieved by changing the overall mean luminance in logarithmic steps of 2. In the other case, motion noise was simulated by presenting moving dots, a certain percentage of which moved coherently first along the preferred and then along the null direction of the motion detector, whereas the remaining dots moved randomly into any other direction ([9]; Fig 4F). The noise susceptibility was quantified as the signal-to-noise ratio of the detector response, i.e. the difference between the average output signals during preferred and null direction simulation, divided by the square root of the response variance.

Such a motion detector revealed a strong directional selectivity over a large range of temporal frequencies (Fig 4B and 4C, green traces): With a peak of the preferred direction response at 2 Hz, the model responded virtually not at all during null direction motion. It also showed a narrow directional tuning (Fig 4D, green trace) with a response amplitude falling to less than 50% for gratings drifting 60 deg away from its preferred direction. For photon noise, the signal-to-noise ratio of the motion detector declines gradually over two orders of magnitude, from about 100 to 1 (Fig 4E, green trace). For motion noise, the signal-to-noise ratio is already at values above 1 at 20% coherence reaching a maximum value of almost 1000 at 100% coherence (Fig 4F, green trace). In order to assess the contribution of each mechanism individually, model simulations were repeated with either the left or the right arm blocked (Fig 4A, middle: Null direction suppression = NDS only, brown traces. Fig 4A, bottom: Preferred direction enhancement = PDE only, orange traces). For gratings drifting along the preferred direction, the model with only NDS reached maximum responses at higher temporal frequencies, whereas the model with only PDE performed in an almost identical way to the full model (Fig 4B). Both partial models showed significant responses to gratings drifting along the null direction (Fig 4C). With respect to their directional tuning, the model with only NDS revealed some broadening compared to the full model, while the model with only PDE performed significantly worse (Fig 4D). When confronted with photon noise, the signal-to-noise ratio of both partial models dropped more steeply with increasing input noise than the full model, with the model relying only on null direction suppression performing better than the one relying only on preferred direction enhancement (Fig 4E). In a similar way, both partial models turned out to be less sensitive to the number of dots moving coherently (Fig 4F).

**Fig 5:**
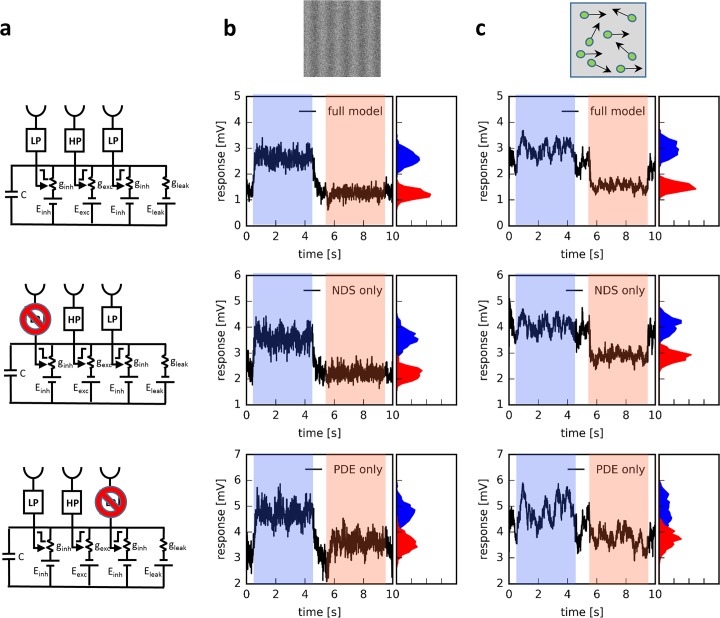
Comparison of noise sensitivity of the full model with both partial models. **a** Circuit diagrams of all three detector models. **b** Responses to a sine grating moving in the preferred direction (PD, from 0.5 sec to 4.5 sec) and the null direction (ND, from 5.5 sec to 9.5 sec) of the detector. To the right in each panel, the signal distributions during both stimulus periods are shown as histograms. In this example, the noise amplitude corresponded to a mean luminance of 4. **c** Same as b, but instead of a noisy sine-grating, the detectors were stimulated by moving dots where 80% of the dots were moving coherently along the preferred (first stimulus period) and null direction (second stimulus period), while 20% of the dots moved into randomly chosen directions.

What is the reason for the full model to exhibit a higher signal-to-noise at its output, compared to both partial models, when confronted with noisy input? In order to investigate this question, the responses of all three models (Fig 5A) are shown in the presence of photon noise (Fig 5B, mean luminance level = 4) and in response to moving dots (Fig 5C, coherence level = 80%). As can be seen in the histograms, shown next to the time-dependent response traces, the response variances, i.e. the widths of the response distributions, are somewhat smaller for the full model than for each of the partial models, both during preferred and during null direction motion. This is due to the fact that both partial models share the central input which, due to its high-pass properties, is more noise-sensitive than the left and the right input signals which are low-pass filtered. This way, the interaction between both mechanisms can reduce the noise more effectively than each partial model alone. In addition, the difference between the mean preferred direction and null direction response is larger for the full model, as compared to both partial model. Both effects lead to an increase of the signal-to-noise ratio. In summary, a motion detector that implements both preferred direction enhancement and null direction suppression in a biophysically plausible way by passive ionic conductances reveals a strong direction selectivity, narrow directional tuning and turns out to be rather noise insensitive. This behavior is not attributable to any one of the two mechanisms alone but rather rests on the combination of both of them.

## Discussion

How neurons in the visual system compute the direction of motion from non-directional input signals, i.e. the emergence of direction selectivity, has been a prime example for neural computation in general for long and a field of intense studies in both vertebrates and invertebrates (for review, see [16]). In the fruit fly *Drosophila*, our current understanding has reached a level where not only the exact location of this computation is known, i.e. the dendrite of T4 cells in the ON pathway, but also the identity and visual response properties of the input neurons, their transmitter phenotype as well as their precise placement on the dendrite. This opens the door to ask for the biophysical implementation of the underlying computations. At an algorithmic level, these computations comprise a signal amplification for motion along the preferred direction and a signal suppression for motion along the null direction of T4 cells, with both mechanisms at work at different locations within the receptive field [47–49]. Null direction suppression can be implemented by an inhibition opening a Chloride conductance several times larger than the leak conductance (‘shunting inhibition’, [56]) or, alternatively, by a modest inhibitory conductance change followed by rectification. The latter would reflect the voltage threshold of a Calcium channel. In contrast to null direction suppression, preferred direction enhancement seems more complex to understand in biophysical terms. Here, several proposals have been made in the past (reviewed in [55]) that include simple threshold nonlinearities [57], log-exp transforms exploiting the relation of *x* ⋅ *y* = exp(*log x* + *log y*) [22], NMDA receptors and chemical cooperativity. In contrast to most of the above, the mechanism advocated here relies purely on passive membrane properties of the postsynaptic neuron. Inspired by the fact that the input neuron in place for signal amplification has an OFF center receptive field and is potentially inhibitory, the proposed mechanism involves a decrease of inhibition for preferred direction signals leading to subsequent signal enhancement via an increase of the postsynaptic input resistance (see also [55]). As is shown by a variety of tests (Fig 4), a motion detector employing such a mechanism leads to substantial degree of direction selectivity even in the absence of null direction suppression. It does so within a physiological range of conductance changes that are always smaller or at most equal to the resting conductance. The advantage of this mechanism over others relying purely on thresholds or other types of output nonlinearities implemented by the supra-linear behavior of voltage-gated ion channels lies in the fact that signal amplification is obtained over a large range of input signal amplitudes, as are expected to occur in natural environments (see Fig 2H).

Using membrane potential recordings from T4 cells together with apparent motion stimulation, a recent publication reports null direction suppression only, with no sign of preferred direction enhancement [46]. At first sight, this seems to be in conflict with several Calcium imaging studies that reported clear signs of preferred direction enhancement underlying direction selectivity in T4 cells [44,45,47–49]. This discrepancy could be explained by the action of a voltage-activated Calcium channel leading to a supralinear increase of Calcium concentration based on a current that is too small to be detectable in voltage recordings. A more likely explanation relies on the observation that null direction suppression is more sensitive at smaller stimulus sizes or intensities than preferred direction enhancement (see Fig 2F in [48]). Based on these different sensitivities, one expects no preferred direction enhancement at 2 deg bar width as was used in Gruntman et al [46]. Obviously, more experiments are required to distinguish between these alternatives and to answer the fundamental question whether the membrane potential behaves linearly and supralinearity is expressed only at the level of the Calcium concentration. Apart from that, the specific role of the different input neurons to T4 cells can be tested by permanent blocking [58], optogenetic hyper- [59] or depolarization [43] as well as removal of postsynaptic transmitter receptor via RNAi techniques [43] or genome editing [53,60,61]. Given that many models of motion detection imply a multiplicative-like interaction (see Introduction), a detailed understanding of the biophysical mechanism underlying preferred direction enhancement in the fly T4 neuron will be of general interest beyond fly motion vision.

## Methods

### Stimulus generation

Stimulus movies were generated as an array of 200 x 200 pixels and 1000 time points corresponding to 180 x 180 degree of visual space and 10 seconds of time. Sine-gratings had a spatial wavelength of 36 degree, an average luminance of 0.5 and a modulation from 0 to 1, i.e. the contrast was 100%. Photon noise was simulated as shot noise by drawing random numbers from a Poisson distribution with the mean value λ proportional to the value of each image pixel I(x,y,t) of the sine grating. Different noise levels were achieved by different overall mean levels of luminance, i.e. by multiplying the image with a given factor (1,2,4,8,16,32) before drawing values from the Poisson distribution. Subsequently, all images were normalized to have the same mean luminance and contrast. Motion noise was generated by randomly placing 500 dots of 1 pixel size onto the visual scene. A certain percentage of these dots moved coherently into one direction while the rest moved into a random direction, which changed every 50 msec. Subsequently, the stimulus movie was spatially low-pass filtered by a Gaussian function of 4.5 degree half-width.

### Motion detection

First, the visual input was down-sampled to an array of 40 x 40 photoreceptors. This way, each photoreceptor received input from 4.5 x 4.5 degree of visual space. Next, the signal was high-pass filtered with 250 msec time-constant. 10% of the photoreceptor signal was added to the output of the filter. In the ON pathway, this signal was rectified at 0. In parallel, the photoreceptor signal was also low-pass filtered with a time-constant of 50 msec. In the OFF pathway, the high-pass signal was sign-inverted and rectified at 0, the low-pass signal was obtained by low-pass filtering 1 minus the photoreceptor signal. These filtered signals were then passed onto an array of 38 x 40 local motion detecting units simulating T4 cells tuned to rightward motion. Each such unit received as its left input, corresponding to the Mi9 cell, the low-pass signal from the OFF pathway and as its central and right input, corresponding to the Mi1 and Mi4 cell, the high-pass and the low-pass signal from the ON pathway, respectively. The excitatory conductance g_exc_ was set to the Mi1 signal, the inhibitory conductance g_inh_ was set to the sum of the Mi9 and Mi4 signals. The resulting membrane potential was then calculated as $V_{m}=\frac{E_{exc}g_{exc}+E_{inh}g_{inh}}{g_{exc}+g_{inh}+g_{leak}}$ with *E*_*exc*_ = +50 *mV*, *E*_*inh*_ = −20 *mV*, *g*_*leak*_ = 1. These signals were spatially averaged after rectification at 0 mV. All simulations were written in Python. The software is available as supplemental information.

## Supporting information

S1 File Python library of routines used by all programs. (PY)

S2 File Source code to generate the simulation results shown in Fig 2. (PY)

S3 File Source code to generate the simulation results shown in Fig 3. (PY)

S4 File Source code to generate the simulation results shown in Fig 4. (PY)

S5 File Source code to generate the simulation results shown in Fig 5. (PY)
